# Machine learning for tissue diagnostics in oncology: brave new world

**DOI:** 10.1038/s41416-019-0535-1

**Published:** 2019-08-09

**Authors:** Niels Halama

**Affiliations:** 1 Department of Medical Oncology and Internal Medicine VI, National Center for Tumor Diseases, University Hospital Heidelberg, Heidelberg, Germany; 2 German Translational Cancer Consortium (DKTK), Heidelberg, Germany; 3 Institute for Immunology, University Hospital Heidelberg, Heidelberg, Germany; 4 Department of Translational Immunotherapy, German Cancer Research Center (DKFZ), Heidelberg, Germany; 5 Helmholtz Institute for Translational Oncology (HI-TRON), Mainz, Germany

**Keywords:** Cancer microenvironment, Machine learning, Computer science

## Abstract

Machine learning is an exciting technology with broad application in big data analysis, as well as increasingly in specialised healthcare. As a diagnostic tool in tissue workup and pathology, it has the potential for personalised and stratified approaches, but the limitations and pitfalls need to be better understood and characterised especially in this critical area of medical care.

## The beginning: learning machines in medicine

The development of powerful algorithmic approaches, termed ‘machine learning’, closely follows the development of modern computer technology. The promises of machine learning in medicine revolve around the notion of faster and more reliable classification of images or datasets. Especially in oncology, the possible applications are boundless: from the classification of imaging studies (‘tumour’ versus ‘no tumour’) to the classification of cells within a tissue section. The roots of machine learning lie within the conceptional beginning of circuit design: algorithmic investigations have held a tight grip on how data were perceived and understood. One area that is now attracting more and more research interest is the analysis of tissue specimens—harvesting more information from ‘pure’ tissue sections, i.e. tissue material processed in standardised routine procedures and available from large numbers of patients. Tissue diagnostics and processing is the field of work of the pathologist, and it is not visionary to predict that image analysis and machine learning will further shape the way pathologists will work in the future.

## The new ‘microscope’ for tissue: computational tools develop with computational power

Tissue specimens, especially those processed and subjected to haematoxylin-eosin staining, are available in large quantities from a large number of oncological patients. Images generated from these large sections with routine counterstains offer rich information. Fundamental aspects of cellular composition, localisation and quantity can be gained from these images. Without specific staining procedures, it is difficult for the human eye to identify the subsets of cells and to precisely quantify these subsets robustly. There are clear examples where one can expect advantages from a computerised approach: lymphocyte infiltration is a good prognostic factor in many tumour entities. Dataset size is an important factor in machine learning: datasets beyond 1000 data points of uniform type are usually needed for creating robust predictors. With the advent of whole slide image scanners for histology, the availability of large patient datasets (of larger numbers) has increased even more. The type of machine learning algorithms applied to these medical images has developed over time, and the complexity of these algorithms ranges from single-layer neural nets to complex deep learning (Boltzmann) algorithms. The history of machine learning is winding, with key figures in the 50s and 60s of the last century being Marvin Minsky, Frank Rosenblatt and Charles Wightman.^1^ In this evolution, convolutional neuronal networks (CNN)^2^ have provided a significant, new, and technically efficient approach.

With this technical advancement, more and more far-reaching classifications and stratifications have been attempted with machine learning. Aligning the treasure chests of ‘big data’ with clinical outcomes has been also in the focus of attention, chemotherapy response prediction in colorectal cancer patients being just one example.^3^ With the focus on tissue, the identification of predictive features within the tissue section was performed^4,5^ (including lymphocytes or vasculature).^6–8^ A good example is the identification of immunohistochemistry-based signatures to predict metastatic sites of triple-negative breast cancers.^8^ Finding ‘unseen’ aspects in tissue sections to align genetic alterations with phenotypic features is also a key aspect of new developments.^9,10^ However, with this advancement, especially for medical application on tissue, new fields of problems have appeared.

## Brave new world: a (computational) stratification tool is still a stratification tool

The prerequisite for successful machine learning approaches is still a sufficient dataset size. This is clearly limiting the use of this technology, because the low frequency of certain cancer entities limits available material. This also leads to the misinterpretation of exploratory analyses and points to a need for extensive validation. This is not to be disregarded in a computational approach, which might be easy to transfer from one institution to another. Validating the possible diagnostic machine learning approach requires the same tight controls and quality assurance management as any other medical validation approach with wet lab technology.

**Fig. 1:**
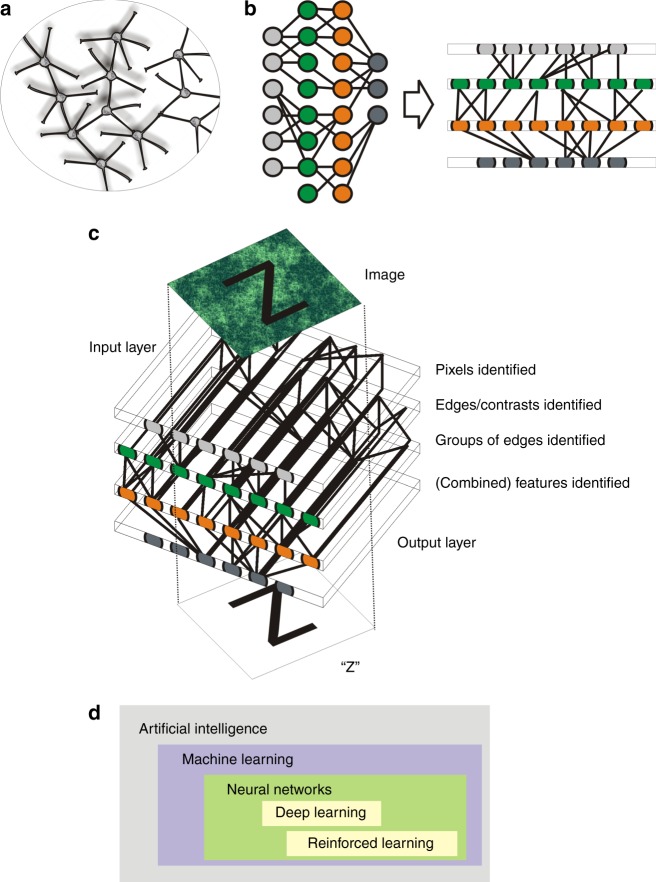
Biology-inspired (**a**) design of circuits led to the development of digital neural networks (**b**), coupling different layers to form a structure for feature recognition, separating an input layer and inner layers from the output (**c**), with variations in the design of the networks leading to an evolution of different applicability and technical parameters (**d**)

Another important point here is to understand the predictive features within the tissue (or the image, see Fig. 1). One way is obscuring the features within the image systematically to identify elements that inform the predictive algorithm. This also opens the door to understand ‘what precisely’ the machine learning algorithm sees in the tissue, e.g. lymphocytes (i.e. round cells without significant cytoplasm). Possible confounders or bias can be identified as well, e.g. the counterstain. Here, the definition of ‘interpretability’ is important—translating the algorithmic findings into human-understandable language or symbols (see e.g. https://fatconference.org/2019/). Missing evidence-based expectations for clinically acceptable performance is another specific danger in machine learning—in other words, the alignment of expected performance with realistic clinical expectations and the validation of it. *Exemplum crudelitatis*, the written clinical annotation on an image as a predictor of outcome and not the actual medical image itself (see https://medium.com/@jrzech/), is a flamboyant example, but one that emphasises quality control and understanding of the algorithm as important parameters of success.

## The outlook: algorithmic tools for pathology

An ideal scenario for development and validation of prediction models should take the abovementioned points into account. Machine learning is best suited for multisite studies and model testing on subsets,^11^ but its application in study design and reporting in medical research requires the development of clearer standards. Algorithmic tools are indeed becoming a part of the armamentarium in tissue diagnostics and pathology, regardless of whether deep learning, multiple-agent simulations or other computing approaches are used.^12^ The advent of another tool in the medical toolbox is always exciting,^13^ but also requires a careful analysis of the tool's boundaries and limitations. Artificial intelligence critic Kate Crawford sums it up: ‘Machine learning does not produce inscrutable and unquestionable objects of mathematics that produce rational, unbiased outcomes. It is human design behind it’. There is no doubt that machine learning will enrich the diagnostic capabilities of pathologists and other medical specialties, but only if mastered properly by trained computer specialists and physicians alike. Medicine needs to shape its tools and not the other way around.

## Data Availability

Not applicable.
